# A pilot study evaluating the feasibility, safety, and efficacy of transcranial photobiomodulation (tPBM) for the treatment of mild cognitive impairment (MCI): preliminary findings

**DOI:** 10.1002/alz.094682

**Published:** 2025-01-09

**Authors:** Neda Rashidi‐Ranjbar, Nathan W. Churchill, Raphael Schneider, Tom A. Schweizer, Corinne E. Fischer

**Affiliations:** ^1^ Keenan Research Centre for Biomedical Science, Li Ka Shing Knowledge Institute, St. Michael’s Hospital, Canada, TORONTO, ON Canada; ^2^ Keenan Research Centre for Biomedical Science, Li Ka Shing Knowledge Institute, St. Michael’s Hospital, Toronto, ON Canada; ^3^ Temerty Faculty of Medicine, Division of Neurology, University of Toronto, Toronto, ON Canada; ^4^ Temerty Faculty of Medicine, Division of Neurosurgery, University of Toronto, Toronto, ON Canada; ^5^ Temerty Faculty of Medicine, Department of Psychiatry, University of Toronto, Toronto, ON Canada

## Abstract

**Background:**

Mild Cognitive Impairment (MCI) serves as a precursor to Alzheimer’s dementia (AD). Recent research underscores the relationship between mitochondrial dysfunction and amyloid beta accumulation, underscoring the prospect of targeting mitochondrial function for intervention. Consequently, our study aimed to explore the efficacy of transcranial photobiomodulation (tPBM), a novel non‐invasive technique utilizing near‐infrared light to activate mitochondrial cytochrome C oxidase receptors, thereby enhancing cellular energy in individuals with MCI.

**Methods:**

Fifteen MCI patients were randomly allocated to either active or sham groups (8 active, 7 sham), with both groups receiving indistinguishable active and sham tPBM devices. Over a span of 6 weeks, participants underwent daily home‐based tPBM sessions. Pre‐ and post‐treatment assessments included a comprehensive battery of tests, encompassing the Trail Making Test (TMT B & A), Mini‐Mental State Examination (MMSE), Proton Magnetic Resonance Spectroscopy (H‐MRS) of the posterior cingulate cortex, structural MRI, resting‐state functional MRI (rsfMRI), and blood analyses.

**Results:**

In the active treatment group compared to the sham group, significant findings (p<0.05) emerged including: 1) faster executive functioning (reduced TMT B completion time), and a promising trend of enhanced MMSE and TMT B/A scores. 2) H‐MRS analysis revealed a decreased ratio of N‐acetyl aspartate to creatine (NAA/Cr), indicating improved neuronal health. 3) Structural MRI showed increased volume of the right thalamus. 4) Functional connectivity analysis using rs‐fMRI data unveiled higher change in the default mode network (DMN) and limbic network, as well as between DMN and executive control network (ECN). 5) Blood tests showcased decreases in isoleucine, methionine, and sarcosine levels—markers linked to AD and amyloid plaque formation—while displaying increases in butyrate, L‐carnitine, and L‐arginine levels—markers indicative of improved mitochondrial function. Furthermore, a notable trend towards decreased levels of tau protein was observed.

**Conclusions:**

Our preliminary findings suggest a potential therapeutic benefit of tPBM in MCI, possibly through the augmentation of mitochondrial function. However, extending the treatment duration may yield further improvements in cognition. Parameters such as NAA/Cr ratios via H‐MRS, DMN functional connectivity via rs‐fMRI, and blood metabolites may serve as objective indicators of response to tPBM treatment. Nevertheless, conclusive determinations require a larger sample size.

